# Mode of birth and maternal depression/severe anxiety: Findings from Millennium Cohort Study

**DOI:** 10.1371/journal.pone.0327129

**Published:** 2025-06-27

**Authors:** Elizabeth O. Bodunde, Fergus P. McCarthy, Karen O’connor, Karen Matvienko-Sikar, Ali S. Khashan

**Affiliations:** 1 School of Public Health, University College Cork, Cork, Ireland; 2 INFANT Research Centre, University College Cork, Cork, Ireland; 3 Department of Obstetrics and Gynaecology, Cork University Maternity Hospital, Cork, Ireland; 4 RISE, Early Intervention in Psychosis Team, South Lee Mental Health Services, Cork,; 5 Department of Psychiatry and Neurobehavioral Science, University College Cork, Ireland; Zurich University of Applied Sciences: ZHAW Zurcher Hochschule fur Angewandte Wissenschaften, SWITZERLAND

## Abstract

**Introduction:**

Limited evidence exists on the association between mode of birth and long-term depression and/or severe anxiety in mothers. We aimed to examine the association between mode of birth and depression and/or severe anxiety by 14 years postpartum.

**Methods:**

We used data from the Millennium Cohort Study. Data on mode of birth were collected when mothers were 9 months postpartum, and categorized as spontaneous vaginal birth (VB), assisted VB, induced VB, emergency cesarean section (CS), planned CS, and CS after induction. Depression/severe anxiety were collected as one variable and self reported by mothers at 9 months, 3, 5, 7, 11, and 14 years postpartum based on a doctor diagnosis. The primary outcome measure was a diagnosis of depression/severe anxiety up to 14 years postpartum. We used multivariable logistic regression models to estimate crude and adjusted odds ratios (OR) for the association between mode of birth and depression/severe anxiety by 14 years postpartum.

**Results:**

There were 10,507 singleton mothers included in our analyses. Fully adjusted odds ratio (aOR)for the association between mode of birth and depression/severe anxiety by 14 years postpartum was induced VB, (aOR, 1.13 [95% CI], 1.01–2.28), assisted VB (aOR, 1.03 [95% CI], 0.89–1.19), Emergency CS, (aOR, 1.08 [95% CI], 0.92–1.27), planned CS (aOR, 1.09 [95% CI], 0.93–1.27), and CS after induction (aOR, 1.08 [95% CI], 0.91–1.28). Fully adjusted models did not report any significant association between mode of birth and depression/severe anxiety at other postpartum time points.

**Conclusions:**

The present findings provide support for association between induction of labor and the risk of long-term depression/severe anxiety by 14 years postpartum. The findings provide no evidence to support association between other modes of birth and maternal depression/anxiety.

## Introduction

The rate of obstetric interventions, including cesarean sections (CS) has shown a notable increase over the past three decades with variations across different countries [1,2]. This increase has been the highest in Eastern Asia (44.9%), Western Asia (34.7%), and Northern Africa (31.5%) [1]. While most births are spontaneous vaginal births (VB), CS is recognized as a safe surgical procedure and may be recommended in the event of pregnancy or birth complications [3,4]. Although CS is acknowledged as a vital lifesaving procedure, they are not free of risk and may be associated with maternal postpartum morbidity compared to vaginal birth [5–7]. Recent studies have reported that CS and other interventions such as operative vaginal birth using forceps have been linked to postpartum haemorrhage and pelvic floor disorders compared to vaginal birth [8,9]. In a systematic review, mode of birth was recognized as an important determinant of postpartum health problems such as mood disorders, anxiety disorders, and post-traumatic stress disorders [10].

The link between the mode of birth and mental health problems such as depression and anxiety in the first year after childbirth (the perinatal period) has been extensively studied [11–13]. Perinatal depression and anxiety disorders are known to have a substantial impact on the mother’s mood, sleep pattern, interactions with partner, family as well as on the long-term cognitive development of infants [14]. Studies have consistently demonstrated comorbidity between depression and anxiety disorders [15]. An Australian study also reported a significant overlap between major depression and anxiety disorders in postnatal women [16]. Depression affects up to 20% of healthy mothers between 7–12 months postpartum [17]. Perinatal anxiety disorders are also common; they can be co-morbid with depression or occur independently with a prevalence ranging from 4 to 20 percent [18]. However, their prevalence beyond the customary perinatal period has been less studied, and little is known about the long-term impact of mode of birth. Existing evidence is limited, and large population-based studies have provided inconsistent results [19–21]. Henderson and Quenby’s study indicated that mothers who delivered via CS may be at a higher risk for depression at 3 years postpartum [20]. On the other hand, a systematic review and meta-analysis including 43 studies found no significant difference between CS and VB in the risk of severe depression at 16 months postpartum [22].

The inconsistencies observed in these existing studies may be explained by factors such as the type of CS (elective vs emergency), differences in how depression and anxiety are measured (self-reported vs physician diagnosed), different follow-up periods, small sample sizes, and the quality of data and methods used [23]. The inconsistencies observed in these existing studies may be explained by factors such as the type of CS (elective vs emergency), differences in how depression and anxiety are measured (self-reported vs physician diagnosed), different follow-up periods, small sample sizes, and the quality of data and methods used. Limited evidence exists on other modes of birth such as planned vs emergency CS, assisted, induced vs spontaneous VB. In addition, very few studies account for potential factors that may modify the association such as pre-existing mental disorders [21], the presence of longstanding illnesses in mothers or children [24], and complications in pregnancy or birth. Therefore, we aimed to explore the association between mode of birth and depression/severe anxiety in the mother by 14 years postpartum.

## Materials and methods

### Study design

**Fig 1:**
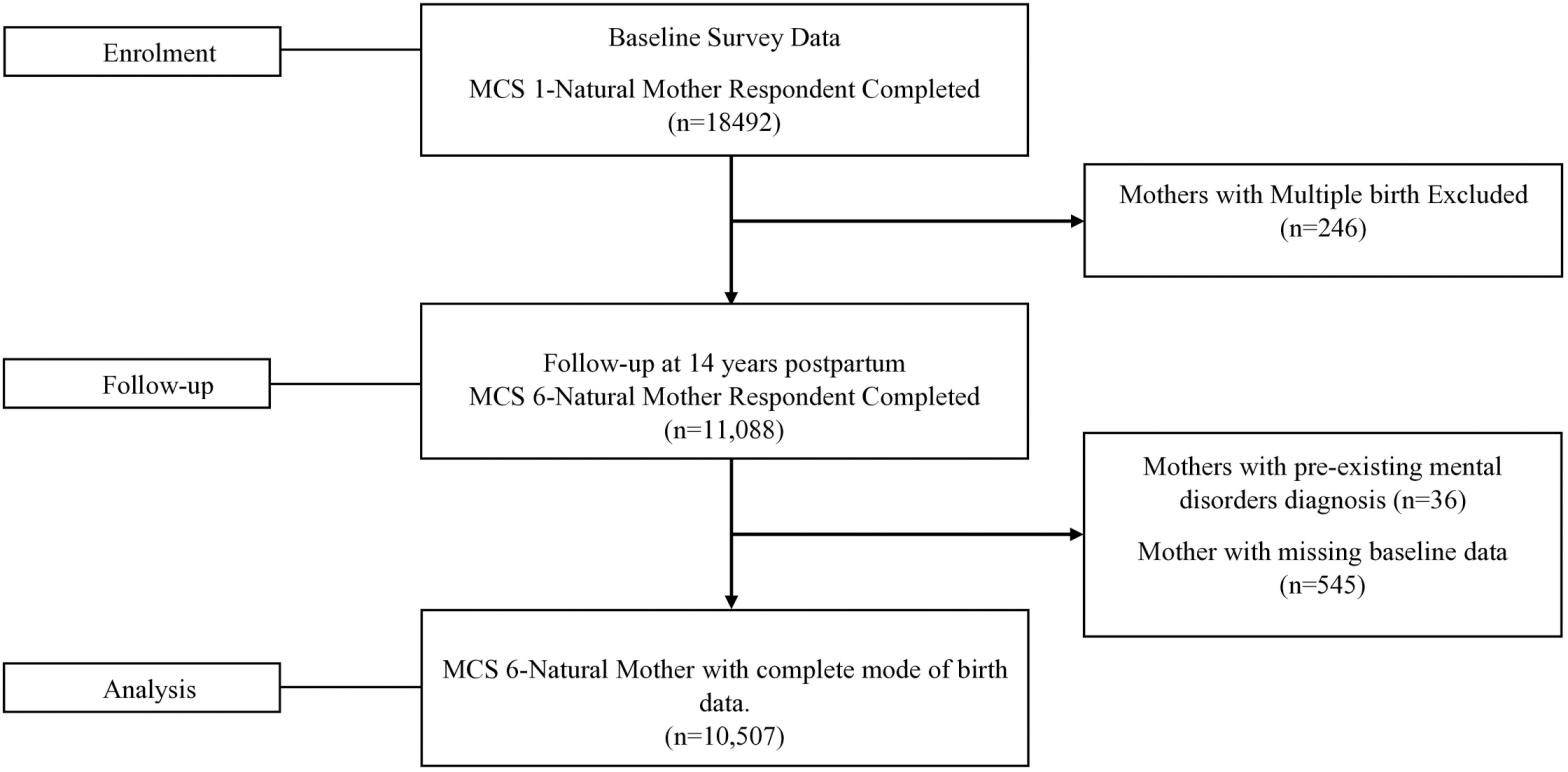
Flowchart of sample determination.

We conducted a secondary analysis of the Millennium Cohort Study (MCS) following the Strengthening the Reporting of Observational Studies in Epidemiology (STROBE) reporting guidelines. The MCS is a nationally representative prospective cohort study of mothers who gave birth between 2000 and 2002 in Great Britain and Northern Ireland. The study data design and collection have been described elsewhere in detail [25]. In summary, the MCS sample was obtained from electoral wards across the UK and was clustered geographically to achieve an adequate representation of the UK population. The baseline (MCS1) data collection occurred when mothers were 9 months postpartum. There have been six subsequent sweeps of data collection at 3 years postpartum (MCS2), 5 years postpartum (MCS3), 7 years postpartum (MCS4), 11 years postpartum (MCS5), 14 years postpartum (MCS6), and 17 years postpartum (MCS7). The MCS recruited 18,492 biological mothers in the baseline survey interviews, of which 11,088 participated in the follow-up at 14 years postpartum (60.8%). The focus of the 17-year postpartum sweep was the child and not the mother so not included in this current study. We excluded mothers if they had multiple births, pre-existing mental disorders, and missing data on mode of birth. Our final sample (10,507) included singleton mothers with complete mode of birth data who provided data on depression/severe anxiety at all relevant time points (Fig 1).

### Exposure and outcomes

Mode of birth was recategorized into (1) spontaneous VB [reference group], (2) assisted VB, (3) induced VB, (4) emergency CS, (5) planned CS, and (6) CS after induction of labor. Mode of birth was considered ‘spontaneous VB’ if mothers had a ‘normal delivery’ or ‘water birth’ and responded ‘no’ to the question ‘was the labor induced or attempted to be induced’. Mothers who had a ‘normal delivery’ or ‘water birth’ but responded ‘yes’ to the question ‘was the labor induced or attempted to be induced’ were considered ‘induced VB‘. Mothers who reported ‘assisted with forceps’, ‘assisted vacuum extraction’, ‘assisted breach’, or ‘other assisted delivery’ were considered ‘assisted VB. Mothers who responded with ‘emergency CS’ or ‘planned CS’ were recorded as such. Mothers who reported having any CS (emergency or planned) but responded ‘yes’ to the question ‘Was the labor induced or attempted to be induced’ were considered ‘CS after induction’. Answers that recorded ‘other’ or ‘irrelevant’ were considered missing. Data on depression/severe anxiety were collected as a single composite variable. Mothers were asked at every time point if they had ever received a diagnosis of depression/severe anxiety from a doctor. The primary outcome was self-reported diagnosis of depression or severe anxiety at any time point up to 14 years postpartum. We considered secondary outcomes as depression/severe anxiety diagnosis at each time point. A short description of co-variates included in the analysis is provided in S2 Appendix.

### Statistical analysis

Data analyses were performed using Stata/SE v.16 (Stata Corporation, College Station, TX, U.S.A.). First, we conducted a descriptive analysis of the participant’s characteristics according to the mode of birth and assessed the distribution of the mode of birth using Chi-square tests. Following this, we estimated the association between mode of birth and cumulative depression/severe anxiety up to 14 years postpartum by presenting crude and adjusted odds ratios (ORs) with 95% confidence intervals (CIs) using multivariable logistic regression. We performed four logistic regression models. The first model presented crude unadjusted estimates. In the second model, we adjusted for sociodemographic factors (maternal age, ethnicity, and maternal BMI). In the third model, we adjusted for additional factors (area-level deprivation, maternal education, HDP, presence of a longstanding illness in mothers, and parity). The final model presented fully adjusted estimates controlling for factors in the second and third models.

We undertook a range of prespecified subgroup analyses to explore key findings. We stratified analysis by the presence of postnatal psychological distress at 9 months, and NICU admission. We considered only primiparous mothers to eliminate the possibility of potential confounding effects of prior pregnancy or previous mode of birth. We stratified mothers of children born preterm (born before 37^th^ weeks gestation). We stratified mothers by longstanding illnesses as pre-existing morbidity may increase the likelihood of a cesarean birth. In addition, we performed the following sensitivity analyses; First, we categorized the mode of birth into “any cesarean birth” and “any induction” to investigate if different modes of intervention influenced the results. Second, we repeated analyses for our primary outcome for mothers with complete data on mode of birth, depression/severe anxiety, and co-variates. Third, we repeated the main analysis for mothers who were followed up at 14 years postpartum only. For missing data, we coded relevant items as ‘missing’ instead of fitting the model to observations with complete data or using multiple imputations. Overall, statistical significance level was set at *p* < 0.05.

### Ethics statement

The Centre for Longitudinal Studies obtained ethical approval from the South West Multi-Centre Research Ethics Committee (MREC), London MREC, Yorkshire MREC, and Yorkshire and The Humber – Leeds East MREC at each survey; MCS1: South West MREC (MREC/01/6/19); MCS2 and MCS3: London MREC (MREC/03/2/022, 05/MRE02/46); MCS4: Yorkshire MREC (07/MRE03/32); MCS5: Yorkshire and The Humber-Leeds East (11/YH/0203); MCS6: London MREC (13/LO/1786), MCS7: REC North East–York (REC ref: 17/NE/0341). The MCS obtained informed written consent from parent/ carers of all cohort children prior to participating in the study. For this present study the MCS has been fully anonymised and did not require additional ethics approval or consent for further analysis.

## Results

A total of 10,507 singleton biological mothers who were the main respondents were included in the current study. Of the current study cohort, spontaneous VB was reported by 49.0% (n = 5152) participants, induced VB by 19.3% (n = 2032), assisted VB by 10.0% (n = 1046), emergency CS by 8.5% (n = 752), planned CS by 8.0% (n = 841) and CS after induction by 6.5% (n = 684) participants. About 48% (n = 5022) of the participants had at least one diagnosis of depression/severe anxiety by 14 years postpartum. Compared to other modes of birth, mothers who delivered via spontaneous VB were likely to be multiparous, older at childbirth, have normal BMI, third-level educated, and less likely to have a longstanding illness. Maternal and child characteristics are outlined in Table 1.

**Table 1:** Characteristics of mother and child by mode of birth among Millennium Cohort Study participants (n = 10,507).

Characteristics	Spontaneous VB	Induced VB	Assisted VB	Emergency CS	Planned CS	CS after Induction	P value
Total (%)	5152 (49.0)	2032 (19.3)	1046 (10.0)	752 (8.5)	841 (8.0)	684 (6.5)	
Maternal age, n (%)							
14-19	332 (6.4)	178 (8.8)	69 (6.6)	40 (5.3)	15 (1.8)	28 (4.0)	<0.001
20-29	2420 (47.0)	929 (45.7)	466 (44.6)	326 (43.4)	267 (31.8)	272 (39.8)	
30-39	2297 (44.6)	883 (43.4)	495 (47.3)	359 (47.7)	523 (62.2)	361 (52.8)	
40+	101 (2.0)	42 (2.1)	16 (1.5)	27 (3.6)	36 (4.3)	23 (3.4)	
Maternal BMI, n (%)							
Normal (18.5–24.9 kg/m^2^)	3252 (63.12)	1159 (57.0)	707 (67.6)	420 (55.8)	437 (52.0)	345 (50.4)	<0.001
Underweight (<18.5 kg/m^2^)	674 (13.1)	262 (12.9)	99 (9.5)	99 (13.2)	115 (13.7)	75 (11.0)	
Overweight (25–29.9 kg/m^2^)	904 (17.6)	410 (20.2)	183 (17.5)	156 (20.7)	188 (22.3)	158 (23.1)	
Obese (30–39 kg/m^2^)	322 (6.3)	201 (9.9)	57 (5.5)	77 (10.2)	101 (12.0)	106 (15.5)	
Parity							
Multiparous	2963 (57.5)	1062 (52.3)	180 (17.2)	272 (36.2)	565 (67.2)	177 (25.9)	
Primiparous	2189 (42.5)	970 (47.7)	866 (82.8)	480 (63.8)	276 (32.8)	507 (74.1)	<0.001
Ethnicity							
White	4215 (81.8)	1742 (85.7)	953 (91.1)	611 (81.3)	718 (85.4)	581 (84.9)	<0.001
Others	927 (18.0)	14.0 (15.5)	93 (8.9)	141 (18.7)	122 (14.5)	102 (15.1)	
Missing	10 (0.2)	5 (0.3)	–	–	1 (0.1)		
Education							
None of these	849 (14.5)	379 (18.7)	93 (8.9)	94 (12.5)	125 (14.9)	79 (11.6)	
< O level	518 (10.1)	216 (10.6)	71 (6.8)	74 (9.8)	80 (95)	62 (9.1)	<0.001
O level	1687 (32.7)	690 (34.0)	334 (31.9)	219 (29.1)	263 (31.3)	226 (33.0)	
A level	495 (9.6)	213 (10.5)	130 (12.4)	87 (11.6)	65 (7.7)	64 (9.4)	
Diploma/Higher	1431 (27.8)	478 (23.5)	399 (38.2)	255 (33.9)	281 (33.4)	238 (24.8)	
Other qualification	164 (3.2)	53 (2.6)	18 (1.7)	22 (2.9)	26 (3.1)	15 (2.2)	
Missing	8 (0.2)	3 (0.2)	1 (0.1)	1 (0.1)	1 (0.1)	–	
Deprivation level							
Most deprived	1624 (31.5)	630 (31.0)	218 (20.8)	190 (25.3)	215 (25.6)	163 (23.8)	<0.001
Second decile	1106 (21.5)	461 (22.7)	226 (21.6)	167 (22.2)	190 (22.6)	170 (24.9)	
Third decile	885 (17.2)	335 (16.5)	208 (19.9)	135 (18.0)	155 (18.4)	128 (18.7)	
Fourth decile	720 (14.0)	296 (14.5)	195 (18.6)	126 (16.8)	135 (16.0)	100 (14.6)	
Least deprived	817 (15.8)	310 (15.3)	199 (19.0)	134 (17.8)	134 (17.4)	123 (17.0)	
Longstanding Illness in mothers							
No	4200 (81.5)	1532 (75.4)	838 (80.1)	583 (77.5)	619 (73.6)	516 (75.4)	<0.001
Yes	947 (18.4)	500 (24.6)	208 (19.9)	169 (22.5)	222 (26.4)	168 (24.6)	
Missing	5 (0.1)	–	–	–	–	–	
Hypertensive disorders in pregnancy							
No	4970 (96.5)	1819 (89.5)	954 (91.2)	661 (87.9)	770 (91.6)	565 (82.6)	<0.001
Yes	182 (3.5)	213 (10.5)	92 (8.8)	91 (12.1)	71 (8.4)	119 (17.4)	
NICU admission							
No	1448 (28.1)	677 (33.3)	649 (62.1)	436 (58.0)	266 (31.6)	401 (58.6)	<0.001
Yes	247 (4.8)	143 (7.0)	109 (10.4)	190 (25.3)	99 (11.8)	108 (15.8)	
Missing	3457 (67.1)	1212 (59.7)	288 (27.5)	126 (16.7)	476 (56.6)	175 (25.6)	
Postnatal psychological distress at 9 months							
No	4307 (83.6)	1650 (81.2)	903 (86.3)	631 (83.9)	698 (83.0)	564 (82.5)	<0.001
Yes	625 (12.1)	320 (15.8)	119 (11.4)	95 (12.6)	115 (13.7)	98 (14.3)	
Missing	220 (4.3)	62 (3.0)	24 (2.3)	26 (3.5)	28 (3.3)	22 (3.2)	
Ever diagnosed with depression/severe anxiety							
At 9 months postpartum	1136 (22.1)	535 (26.3)	220 (21.0)	170 (22.6)	215 (25.6)	169 (24.7)	0.001
At 3 years postpartum	1228 (26.3)	553 (30.3)	258 (26.9)	177 (25.8)	229 (29.3)	182 (29.0)	0.017
At 5 years postpartum	222 (6.7)	111 (9.1)	60 (8.5)	43 (8.8)	45 (8.5)	27 (6.4)	0.056
At 7 years postpartum	181 (6.0)	71 (6.4)	41 (6.5)	19 (4.4)	30 (6.3)	14 (3.6)	0.253
At 11 years postpartum	210 (7.4)	89 (8.8)	43 (7.4)	33 (8.1)	32 (7.1)	31 (8.1)	0.802
At 14 years postpartum	1223 (27.7)	591 (33.4)	280 (29.4)	182 (27.6)	223 (30.1)	199 (33.9)	<.001

VB; Vaginal birth, CS; Cesarean section, BMI; Body mass index, NICU; Neonatal intensive care unit, HDP; Hypertensive disorders in pregnancy.

Table 2 presents adjusted results for the association between mode of birth and cumulative depression/severe anxiety by 14 years postpartum. Induced VB was associated with a 13% increase in the odds of having a depression/severe anxiety diagnosis by 14 years postpartum compared to spontaneous VB. The ORs of the association between other modes of birth and cumulative depression/severe anxiety by 14 years postpartum did not reach statistical significance.

**Table 2:** Association between mode of birth and cumulative depression/ severe anxiety by 14 years postpartum.

	No of exposed cases	Model 1 OR (95% CI)	Model 2 OR (95% CI)	Model 3 OR (95%CI)	Model 4 OR (95% CI)
Depression/severe anxiety diagnosis by 14 years postpartum					
Spontaneous VB	2367	Ref	Ref	Ref	Ref
Induced VB	1061	1.29 (1.16-1.42)*	1.22 (1.10-1.35)*	1.17 (1.05-1.30)*	1.13 (1.01-1.26)*
Assisted VB	490	1.04 (0.91-1.18)	1.00 (0.87-1.15)	1.03 (0.90-1.19)	1.03 (0.89-1.19)
Emergency CS	359	1.07 (0.92-1.25)	1.01 (0.93-1.26)	1.03 (0.88-1.21)	1.08 (0.92-1.27)
Planned CS	408	1.11 (0.96-1.28)	1.16 (1.00-1.35)*	1.08 (0.92-1.25)	1.09 (0.93-1.27)
CS after Induction	337	1.14 (0.97-1.34)	1.13 (0.96-1.33)	1.04 (0.87-1.23)	1.08 (0.91-1.28)

OR: Odd ratio, 95% CI: 95% Confidence interval, VB: Vaginal birth, CS: Cesarean section, BMI: Body mass index, HDP: Hypertensive disorders in pregnancy.

Model 1: Unadjusted

Model 2: Adjusted for maternal age, ethnicity, prepregnancy BMI.

Model 3: Adjusted for, area deprivation level, maternal education, HDP, longstanding illness, parity.

Model 4: Fully adjusted for variables in model 3 & 4.

*P-value < .05

Table 3 revealed crude and adjusted associations between mode of birth and depression/severe anxiety at 5 time points (9 months, 3, 5, 7, and 11 years postpartum). In the crude analysis, induced VB and planned CS were associated with a diagnosis of depression/severe anxiety at 9 months postpartum, [OR 1.26, (95% CI, 1.2–1.42)] and [OR 1.21, (95% CI, 1.03–1.44)] respectively. The adjusted model showed that induced VB but not planned CS was associated with depression/severe anxiety at 9 months postpartum [OR 1.15 (95% CI, 1.00–1.28). No other modes of birth were significantly associated with depression/severe anxiety at 9 months postpartum. There was no significant association between mode of birth and depression/severe anxiety at 3 years postpartum across all models. At 5, 7, and 11 years postpartum, after adjusting for all maternal and child characteristics, the association between induced VB and depression/severe anxiety remained statistically significant in the crude models, [OR 1.27 (95% CI, 1.14–1.41)], [OR 1.26, (95% CI, 1.13–1.40)] and [OR 1.27 (95% CI, 1.14–1.41)] respectively. However, adjusted models did not report statistically significant results.

**Table 3:** Association between mode of birth and cumulative depression/ severe anxiety at 9months, 3, 5,7, and 11 years postpartum.

	No of exposed cases	Model 1 OR (95% CI)	Model 2 OR (95% CI)	Model 3 OR (95%CI)	Model 4 OR (95% CI)
Depression/severe anxiety diagnosis at 9 months postpartum					
Spontaneous VB	1136	Ref	Ref	Ref	Ref
Induced VB	535	1.26 (1.12-1.42)*	1.20 (1.07-1.37)*	1.15 (1.00-1.28)*	1.10 (0.97-1.25)
Assisted VB	220	0.94 (0.80-1.11)	0.89 (0.75-1.05)	0.97 (0.82-1.15)	0.95 (0.80-1.13)
Emergency CS	170	1.03 (0.86-1.24)	1.02 (0.84-1.23)	1.00 (0.83-1.21)	1.02 (0.84-1.23)
Planned CS	215	1.21 (1.03-1.44)*	1.20 (1.02-1.43)*	1.12 (0.94-1.34)	1.10 (0.92-1.32)
CS after Induction	169	1.16 (0.96-1.40)	1.11 (0.92-1.34)	1.07 (0.88-1.30)	1.07 (0.88-1.31)
Depression/severe anxiety diagnosis at 3 years postpartum					
Spontaneous VB	1610	Ref	Ref	Ref	Ref
Induced VB	726	1.22 (1.09-1.36)*	1.16 (1.04-1.30)*	1.11 (0.99-1.24)	1.07 (0.96-1.20)
Assisted VB	314	0.94 (0.82-1.10)	0.90 (0.77-1.04)	0.97 (0.83-1.13)	0.95 (0.82-1.11)
Emergency CS	237	1.01 (0.86-1.19)	1.01 (0.85-1.19)	0.98 (0.83-1.17)	1.01 (0.85-1.20)
Planned CS	282	1.11 (0.95-1.30)	1.13 (0.96-1.32)	1.05 (0.90-1.24)	1.05 (0.89-1.23)
CS after Induction	231	1.12 (0.95-1.33)	1.09 (0.92-1.30)	1.04 (0.87-1.24)	1.06 (0.88-1.27)
Depression/severe anxiety diagnosis at 5 years postpartum					
Spontaneous VB	1832	Ref	Ref	Ref	Ref
Induced VB	837	1.27 (1.14-1.41)*	1.21 (1.09-1.35)*	1.15 (1.03-1.28)*	1.11 (1.00-1.24)
Assisted VB	374	1.01 (0.88-1.16)	0.96 (0.84-1.11)	1.02 (0.88-1.18)	1.01 (0.87-1.17)
Emergency CS	280	1.08 (0.92-1.26)	1.07 (0.91-1.26)	1.04 (0.88-1.22)	1.07 (0.90-1.26)
Planned CS	327	1.15 (0.99-1.34)	1.18 (1.02-1.38)*	1.11 (0.94-1.29)	1.11 (0.94-1.30)
CS after Induction	258	1.10 (0.93-1.29)	1.07 (0.91-1.27)	1.00 (0.84-1.19)	1.02 (0.85-1.22)
Depression/severe anxiety diagnosis at 7 years postpartum					
Spontaneous VB	2012	Ref	Ref	Ref	Ref
Induced VB	908	1.26 (1.13-1.40)*	1.20 (1.08-1.33)*	1.14 (1.02-1.27)*	1.10 (0.98-1.22)
Assisted VB	415	1.03 (0.90-1.18)	0.98 (0.85-1.13)	1.02 (0.88-1.18)	1.05 (0.90-1.22)
Emergency CS	299	1.03 (0.88-1.20)	1.03 (0.88-1.21)	0.98 (0.84-1.16)	1.05 (0.87-1.24)
Planned CS	357	1.15 (0.99-1.33)	1.19 (1.02-1.38)*	1.11 (0.95-1.30)	1.13 (0.97-1.33)
CS after Induction	272	1.03 (0.87-1.21)	1.01 (0.85-1.19)	0.93 (0.78-1.10)	0.98 (0.82-1.17)
Depression/severe anxiety diagnosis at 11 years postpartum					
Spontaneous VB	2221	Ref	Ref	Ref	Ref
Induced VB	996	1.27 (1.14-1.41)*	1.20 (1.08-1.34)	1.15 (1.03-1.28)*	1.11 (1.00-1.24)
Assisted VB	458	1.03 (0.90-1.18)	0.98 (0.86 −1.13)	1.02 (0.88-1.17)	1.01 (0.88-1.17)
Emergency CS	332	1.04 (0.89-1.22)	1.05 (0.89-1.22)	1.00 (0.85-1.17)	1.04 (0.88-1.22)
Planned CS	388	1.13 (0.98-1.31)	1.17 (1.01-1.36)*	1.10 (0.94-1.28)	1.11 (0.95-1.29)
CS after Induction	303	1.05 (0.89-1.23)	1.03 (0.88-1.21)	0.95 (0.80-1.12)	0.98 (0.82-1.16)

OR: Odd ratio, 95% CI: 95% % Confidence interval, VB: Vaginal birth, CS: Cesarean section, BMI: Body mass index, HDP: Hypertensive disorders in pregnancy.

Model 1: Unadjusted

Model 2: Adjusted for maternal age, ethnicity, prepregnancy BMI.

Model 3: Adjusted for, area deprivation level, maternal education, HDP, longstanding illness, parity.

Model 4: Fully adjusted for variables in model 3 & 4.

*P-value < .05.

S1 Table presents results for the stratified association between mode of birth and depression/severe anxiety by 14 years postpartum. When restricting the analysis to mothers who had postnatal psychological distress at 9 month, fully adjusted models suggested significant association between induced VB [OR 5.45 (95% CI, 4.00–7.42)], assisted VB [OR 4.32 (95% CI, 2.79–6.67)], emergency CS [OR 4.73 (95% CI, 2.83–7.88)], planned CS [OR 9.63 (95% CI, 5.29–17.5)], CS after induction [OR 5.72 (95% CI, 3.34–9.80)]. Induced VB was associated with increased odds of depression/severe anxiety by 14 years postpartum [OR, 1.23 (95% CI, 1.05–1.43)] among primiparous mothers. In mothers with longstanding illnesses, fully adjusted estimates suggested a significant association between mode of birth and depression/anxiety diagnosis by 14 years postpartum; induced VB [OR, 2.27 (95% CI, 2.22–3.34)], assisted VB [OR, 2.25 (95% CI, 1.87–3.39)], emergency CS [OR, 2.33 (95% CI, 1.68–3.23)], planned CS [OR, 2.79 (95% CI, 2.07–3.75)], and CS after induction [OR, 2.95 (95% CI, 2.09–4.17)]. Only emergency CS [OR 1.75 (95% CI, 1.26–2.43)] was associated with depression/severe anxiety by 14 years postpartum among mothers who had preterm birth. By 14 years postpartum, no association was suggested between mode of birth and depression/severe anxiety in either NICU admission stratum.

Compared with any VB, CS (emergency or planned) was not associated with depression/severe anxiety at any postpartum time points S2 Table. Crude logistic regression models suggested increased odds of depression/severe anxiety among mothers who delivered via induced VB compared to mothers who did not at 9 months postpartum [OR 1.21 (95% CI, 1.10–1.34)], 5 years postpartum, [OR 1.19 (95% CI, 1.09–1.30)], 7 years postpartum [OR 1.17 (95% CI, 1.07–1.28)], 11 years postpartum [OR 1.18 (95% CI, 1.08–1.29)], and 14 years postpartum [OR 1.21 (95% CI, 1.12–1.33)]. However, results from the fully adjusted model did not reach statistical significance, (S3 Table). The results from the complete case analysis were similar to the main analysis, as were the findings when restricted to mothers who were followed up at 14 years postpartum only (S4 and S5 Tables).

## Discussion

There is a dearth of evidence on whether mode of birth affects mothers’ long-term mental health [12]. In this study, we examined the association between mode of birth and depression/severe anxiety diagnosis at six postpartum time points up to 14 years postpartum. After controlling for multiple known confounders, only induced VB was associated with increased odds of depression/severe anxiety by 14 years postpartum. Second, the stratified analyses suggested a significant difference in depression/severe anxiety diagnosis by 14 years postpartum among mothers who had postnatal psychological distress. Third, the association between all modes of birth and depression/severe anxiety diagnosis by 14 years postpartum was evident among mothers who had longstanding illnesses.

### Comparison with previous literature

Our main findings are consistent with two studies that demonstrated no significant association between mode of birth and depression or anxiety [19,21]. A Taiwanese study found no association between modes of birth and depression or anxiety after adjusting for covariates, although the authors did not differentiate between emergency and planned CS [19]. Similarly, in a recent longitudinal study conducted among Danish mothers, there was no difference in depressive symptoms among mothers who only had spontaneous VB compared to mothers who had only CS 11.2 years after their last birth [21]. Other studies have observed associations [12,20]. In one study, mothers reported a higher risk of later treatment for depression/anxiety following elective CS compared to vaginal delivery at 3 years postpartum [20]. Another study suggested higher depression, anxiety disorders, and somatization symptoms among women who had instrumental births compared to women who had vaginal delivery [12]. Our study extends previous studies suggesting that unplanned delivery modes may be linked with developing depression [26,27]. A potential explanation underlying these findings is that unplanned delivery modes may be associated with a negative birth experience which could increase a woman’s risk of developing mental health problems [27]. Two studies suggested that unplanned CS may be associated with a loss of control, unmatched expectations, and trauma which may predispose a woman to developing a later mental health condition [26,28]. Other studies revealed that these modes of birth are linked with unexpected complications during childbirth with increased fear of future pregnancies and a possible elevated risk of depression in the longer term [29,30]. In the UK, unplanned birth modes may be performed with medical indications and risk factors, impacting maternal and fetal outcomes [31]. However, some risk factors for unplanned birth mode may also be medical interventions required for the mother or child’s health.

Among women who had postnatal psychological distress at 9 months, we observed an increased odds of depression/severe anxiety by 14 years postpartum. The finding is perhaps unsurprising, given the established link between postpartum mental disorders and subsequent mental health disorders diagnosis [32–34]. When the severity of postpartum mental disorders is considered, postnatal psychological distress may be linked with developing later mental health disorders. According to a study by Abdollahi et al, women who experienced postnatal depression were twice as likely to have depression 4 years after childbirth [33]. The findings in our study of an association between mode of birth and depression/severe anxiety among mothers with longstanding chronic illnesses is novel. A study by Brown et al reported that more women with chronic physical conditions experienced perinatal mental illnesses [35].

Levels of steroid hormones including progesterone, cortisol oestradiol are suggested to play a role in the development of postpartum mental disorders [36,37]. One study hypothesized that hormones such as oxytocin are deficient and dysregulated from normal labor physiology during cesarean births [38]. A disruption of the hormonal environment that supports maternal bonding after childbirth may increase the childbirth stressor and risk of mental health issues. Psychologically, it has been hypothesized that unplanned interventions during childbirth may increase the risk for probable childbirth-related posttraumatic stress disorders (PTSD) due to its links with sense of fear and loss of control [39]. These experiences have been shown to increase the risk of persistent depression or anxiety symptoms induced by the childbirth experience even years after delivery [40,41]_._ However, concensus on the biological and psychological mechanisms that links mode of birth to adverse maternal mental health remain limited and warrants further examination in future research.

### Strengths and limitations

The results of this study should be viewed in the context of some limitations. First, depression/severe anxiety diagnosis relies on self-reported data and may be subject to recall bias [42]. Second, although comorbid, depression and anxiety are distinct mental disorders. We used a combined diagnosis of depression and severe anxiety. As such, it was impossible to conduct separate analysis for depression and anxiety. Therefore, we were not able to examine the association between mode of birth and depression and anxiety separately. Third, we had data on depression/severe anxiety diagnosis, we had no information on the criteria for the severity of the diagnosis. Therefore, we could not evaluate the overall risks and/or benefits of the mode of birth. Fourth, despite adjusting for potential confounders, the role of residual confounding from unmeasured confounding variables such as previous mode of birth and fear of childbirth cannot be precluded. The absence of data on fear of childbirth may have resulted in biased estimates of the association between mode of birth and long-term maternal depression/severe anxiety. As such, some observed associations may be as a result of underlying psychological vulnerability rather than the mode of birth [43,29]. Lastly, selection bias may have been introduced due to loss of follow-up. Our sample included 18,246 mothers at baseline which reduced to 11,088 at 14 years postpartum (of which 10507 were included in the analysis). As participation can be influenced by several factors, mothers with severe mental illnesses may be more likely to be lost to follow up which may have skewed our findings. However, using the cumulative outcome approach to estimate depression/severe anxiety up to 14 years postpartum increased efficiency regardless of the timing of the diagnosis. These study limitations underscore the need for more rigorous investigations in primary studies and suggest the need for further research to characterize specific mental disorders in relation to the mode of birth.

This study also had several strengths. First, this study used a comprehensive, nationally representative large cohort of women who gave birth in the UK between 2000 and 2002. Second, our adjusted estimates included longstanding illnesses, complications during pregnancy or childbirth, and sociodemographic variables which had not been accounted for in previous studies. Third, our analysis excluded mothers with pre-delivery mental health diagnoses as the presence of a long-term mental health diagnosis may be confounded by previous mental disorders exposures. Fourth, the data allowed us to conduct stratified analyses for explanatory factors that could potentially modify the association such as postnatal psychological distress and longstanding illnesses. Lastly, MCS maternal self-reported mode of birth was reported to be highly reliable with an agreement of 94–98% compared to hospital records [44].

## Conclusions

We observed few associations between induced VB and depression/severe anxiety by 14 years postpartum. The associations were stronger for mothers who had postnatal psychological distress and longstanding chronic illnesses. We did not find evidence of association between other modes of birth and depression/severe anxiety by 14 years postpartum. The magnitude of the observed association between induced vaginal birth and depression/severe anxiety by 14 years postpartum were small, potentially due to residual confounding and the clinical implications may be limited. Future research to understand the underlying mechanisms driving these associations and distinguish between specific mental disorders diagnoses is warranted.

## Supporting information

S1 Appendix STROBE Statement. (DOCX)

S2 Appendix Description of co-variates. (DOCX)

S1 Fig Directed Acyclic Graph. (TIF)

S1 Table Stratified association between mode of birth and cumulative depression/severe anxiety by 14 years postpartum by presence of postnatal psychological distress, admission to NICU, parity, presence of longstanding illnesses, and preterm birth. (DOCX)

S2 Table Association between any CS and cumulative depression/severe anxiety at 9 months, 3,5,7,11, and 14 years postpartum among study participants. (DOCX)

S3 Table Association between any induction and cumulative depression/severe anxiety at 9 months, 3,5,7,11, and 14 years postpartum among Millennium Cohort Study participants. (DOCX)

S4 Table Association between mode of birth and cumulative depression/severe anxiety at 9 months, 3, 5,7, and 11 years postpartum among study participants with complete co-variates data (N = 10456). (DOCX)

S5 Table Crude and adjusted association between mode of birth and cumulative depression/severe anxiety by 14 years postpartum-(only including participants at 14 years postpartum follow-up). (DOCX)
