# Supplementary material for: Mode of birth and maternal depression/severe anxiety: Findings from Millennium Cohort Study
Source: PLoS One. 2025 Jun 27;20(6):e0327129. doi: 10.1371/journal.pone.0327129 (PMC12204560; doi:10.1371/journal.pone.0327129)
Supplement: S2 Appendix — (DOCX) [file pone.0327129.s002.docx]

| S2 Appendix: Description of co-variates | |
| --- | --- |
| Variables | **Description** |
| Maternal age | This referred to the mothers age at the time of birth measured in years and referred to mother’s age at time of birth of child. This was included as a categorical variable; 14-19, 20-29, 30-39 and 40+. |
| Prepregnancy BMI | Mothers were asked about their height and weight just before becoming pregnant. This was calculated as weight in kilograms divided by height in meters squared. BMI and was recategorised as ‘underweight’, ‘normal weight’, ‘overweight’ and ‘obese’. |
| Maternal education | This referred to the highest educational level attained by the mother. This was categorised as none, o level, A/AS/S levels, higher degree, first degree, diploma in higher education, other academic qualifications. |
| Ethnicity | Mothers were asked ‘which of the following do you regard yourself belonging to: White, Chinese, Irish Traveller, Indian, Pakistani, Bangladeshi, Black Caribbean, Black African, Black Other, Mixed ethnic group, Any other ethnic group’. This was recategorised as ‘white’ and ‘other ethnic origins. |
| Area-level deprivation | This was determined using geographically linked data including index of multiple deprivation. It was subcategorised into deciles and ranked from most deprived to least deprived. |
| Parity | This was derived from number of siblings in the household, it was recategorised as a dichotomous variable; first child (yes/no). |
| Hypertensive disorders in pregnancy | Mothers were asked “Did you have any illnesses or other problems during your pregnancy”, if mothers answered “yes” to this question and ticked “raised blood pressure, eclampsia, preeclampsia, toxaemia”, they were considered as having a diagnosis. |
| Longstanding illnesses | Mothers were asked if they had any *longstanding illnesses, disability, or infirmity.* This was categorised as yes/no. |
| Postnatal psychological distress | The Nine-item version of the Rutter Malaise Inventory (RMI) questionnaire was used to determine levels of postnatal psychological distress at 9 months. The questionnaire includes nine dichotomous items (yes/no) offering a score from 0 to 9. The nine-item Malaise Inventory scores was computed by adding the score of all nine items, offering a score that ranges from 0-9. For the current study, postnatal psychological distress was determined by score of 4 points or more as suggested by previous studies. |
