# Supplementary figures and images for: Mode of birth and maternal depression/severe anxiety: Findings from Millennium Cohort Study
Source: PLoS One. 2025 Jun 27;20(6):e0327129. doi: 10.1371/journal.pone.0327129 (PMC12204560; doi:10.1371/journal.pone.0327129)

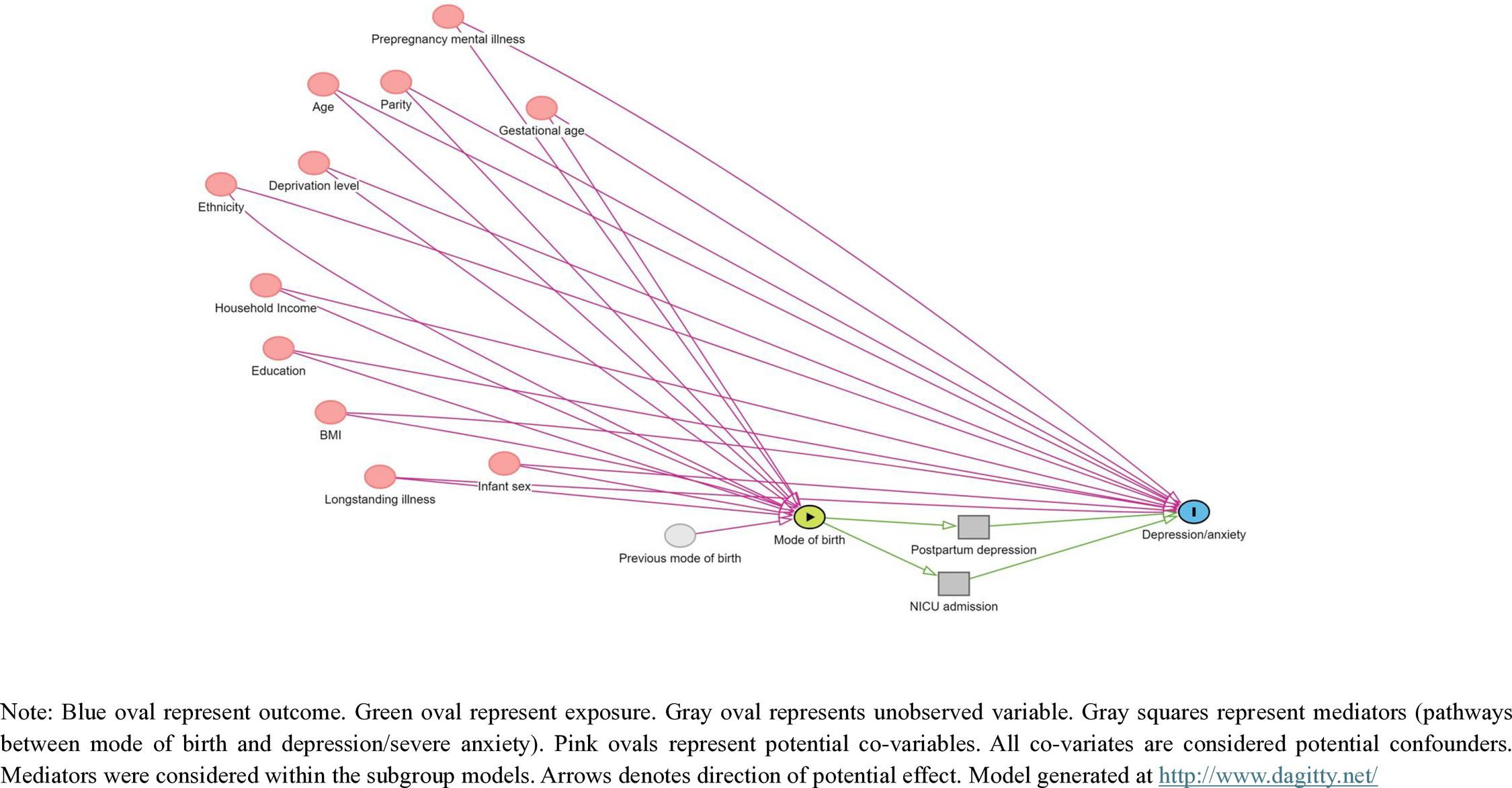

Supplement: S1 Fig — (TIF) [file pone.0327129.s003.tif]
