# Supplementary material for: Mode of birth and maternal depression/severe anxiety: Findings from Millennium Cohort Study
Source: PLoS One. 2025 Jun 27;20(6):e0327129. doi: 10.1371/journal.pone.0327129 (PMC12204560; doi:10.1371/journal.pone.0327129)
Supplement: S1 Table — (DOCX) [file pone.0327129.s004.docx]

| S1 Table: Stratified association between mode of birth and cumulative depression/severe anxiety by 14 years postpartum by presence of postnatal psychological distress, admission to NICU, parity, presence of longstanding illnesses, and preterm birth | | | | | | | |
| --- | --- | --- | --- | --- | --- | --- | --- |
|  | **No of exposed cases** | **OR (95% CI)^a^** | **OR (95% CI)^b^** | **No of exposed cases** | **OR (95% CI)^a^** | **OR (95% CI)^b^** | **P-value for interactions** |
| No Postnatal Psychological Distress at 9 months. | | | | **Postnatal Psychological Distress at 9 months.** | | |  |
| Spontaneous VB | 1786 | **Ref** | **Ref** | 500 | Ref | Ref |  |
| Induced VB | 773 | 1.24 (1.11–1.39)* | 1.10 (0.98–1.24) | 266 | 6.95 (1.11–1.39)* | 5.45 (4.00–7.42)* | 0.789 |
| Assisted VB . | 391 | 1.08 (0.93–1.24) | 1.04 (0.89–1.22) | 88 | 4.00 (2.64–6.05)* | 4.32 (2.79–6.67)* | 0.368 |
| Emergency CS | 272 | 1.07 (0.90–1.27) | 1.05 (0.88–1.26) | 74 | 4.97 (3.05–8.10)* | 4.73 (2.83–7.88)* | 0.607 |
| Planned CS | 2940 | 1.03 (0.87–1.21) | 1.02 (0.85–1.21) | 102 | 11.1 (6.20–19.8)* | 9.63 (5.29–17.5)* | 0.072 |
| CS after Induction | 250 | 1.12 (0.94–1.34) | 1.06 (0.87–1.27) | 80 | 6.27 (3.74–10.5)* | 5.72 (3.34–9.80)* | 0.899 |
|  | **No NICU Admission** | | | **NICU Admission** | | |  |
| Spontaneous VB | 724 | **Ref** | **Ref** | 127 | **Ref** | **Ref** |  |
| Induced VB | 391 | 1.36 (1.14–1.64)* | 1.21 (1.00–1.48) | 83 | 1.38 (0.98–1.95) | 1.23 (0.86–1.78) | 0.868 |
| Assisted VB | 319 | 0.97 (0.80–1.16) | 1.09 (0.88–1.33) | 61 | 1.27 (0.86–1.88) | 1.30 (0.86–1.97) | 0.633 |
| Emergency CS | 204 | 0.88 (0.71–1.09) | 0.96 (0.76–1.20) | 104 | 1.21 (0.89–1.64) | 1.25 (0.90–1.73) | 0.383 |
| Planned CS | 138 | 1.08 (0.83–1.40) | 1.08 (0.82–1.43) | 56 | 1.30 (0.86–1.96) | 1.26 (0.80–1.95) | 0.756 |
| CS after Induction | 199 | 0.99 (0.79–1.23) | 1.04 (0.82–1.32) | 53 | 0.96 (0.65–1.42) | 0.93 (0.61–1.41) | 0.529 |
|  | **Multiparous women** | | | **Primiparous Women** | | |  |
| Spontaneous VB | 1286 | Ref | Ref | 1081 | Ref | Ref |  |
| Induced VB | 530 | 1.30 (1.13–1.49)* | 1.14 (0.98–1.32) | 531 | 1.57 (1.36–1.82)* | 1.23 (1.05–1.43)* | 0.873 |
| Assisted VB | 84 | 1.14 (0.84–1.54) | 1.21 (0.88–1.65) | 406 | 1.15 (0.99–1.34) | 1.07 (0.91–1.25) | 0.226 |
| Emergency CS | 131 | 1.21 (0.94–1.55) | 1.28 (0.99–1.67) | 228 | 1.18 (0.97–1.43) | 1.05 (0.86–1.29) | 0.086 |
| Planned CS | 274 | 1.23 (1.02–1.47)* | 1.17 (0.97–1.42) | 134 | 1.23 (0.96–1.57) | 1.04 (0.81–1.36) | 0.208 |
| CS after Induction | 85 | 1.20 (0.88–1.63) | 1.10 (0.80–1.52) | 252 | 1.29 (1.06–1.55)* | 1.15 (0.94–1.41) | 0.802 |
|  | **Without longstanding illness** | | | **With longstanding illness** | | |  |
| Spontaneous VB | 1760 | Ref | Ref | 603 | Ref | Ref |  |
| Induced VB | 720 | 1.23 (1.09–1.38)* | 1.13 (1.00–1.27) | 341 | 2.97 (2.43–3.62)* | 2.72 (2.22–3.34)* | 0.908 |
| Assisted VB | 356 | 1.02 (0.88–1.19) | 1.04 (0.89–1.22) | 134 | 2.51 (1.88–3.35)* | 2.52 (1.87–3.39)* | 0.918 |
| Emergency CS | 255 | 1.08 (0.90–1.28) | 1.12 (0.93–1.34) | 104 | 2.22 (1.62–3.04)* | 2.33 (1.68–3.23)* | 0.517 |
| Planned CS | 259 | 1.00 (0.81–1.18) | 1.06 (0.90–1.27) | 149 | 2.83 (2.12–3.76)* | 2.79 (2.07–3.75)* | 0.590 |
| CS after Induction | 219 | 1.02 (0.85–1.23) | 1.05 (0.87–1.28) | 118 | 3.27 (2.33–4.58)* | 2.95 (2.09–4.17)* | 0.432 |
|  |  | **Full term birth** | | **Preterm birth** | | |  |
| Spontaneous VB | 2228 | Ref | Ref | 139 | Ref | Ref |  |
| Induced VB | 998 | 1.27 (1.14–1.41)* | 1.12 (1.00–1.25)* | 63 | 1.86 (1.25–2.77)* | 1.44 (0.95–2.19) | 0.277 |
| Assisted VB | 461 | 1.03 (0.89–1.18) | 1.01 (0.87–1.17) | 29 | 1.37 (0.80–2.35) | 1.40 (0.80–2.46) | 0.285 |
| Emergency CS | 252 | 0.92 (0.77–1.09) | 0.93 (0.77–1.12) | 107 | 1.83 (1.35–2.50)* | 1.75 (1.26–2.43)* | 0.004 |
| Planned CS | 378 | 1.10 (0.94–1.28) | 1.08 (0.92–1.26) | 30 | 1.36 (0.80–2.31) | 1.28 (0.73–2.24) | 0.560 |
| CS after Induction | 308 | 1.16 (0.98–1.37) | 1.10 (0.92–1.32) | 29 | 1.04 (0.63–1.72) | 0.87 (0.52–1.51) | 0.521 |
| OR: Odd ratio, 95% CI: % Confidence interval, VB: Vaginal birth, CS: Cesarean section, BMI: Body mass index, HDP: Hypertensive disorders in pregnancy.  ^a^Crude model  ^b^Adjusted for maternal age, ethnicity, prepregnancy BMI, area deprivation level, maternal education, HDP, longstanding illness, parity.  *P-value <.05 | | | | | | | |
