# Supplementary material for: Mode of birth and maternal depression/severe anxiety: Findings from Millennium Cohort Study
Source: PLoS One. 2025 Jun 27;20(6):e0327129. doi: 10.1371/journal.pone.0327129 (PMC12204560; doi:10.1371/journal.pone.0327129)
Supplement: S2 Table — (DOCX) [file pone.0327129.s005.docx]

| S2 Table: Association between any CS and cumulative depression/severe anxiety at 9 months, 3,5,7,11, and 14 years postpartum among study participants. | | | | | |
| --- | --- | --- | --- | --- | --- |
|  | **No of exposed cases** | **Model 1**  **OR (95% CI)** | **Model 2**  **OR (95% CI)** | **Model 3**  **OR (95%CI)** | **Model 4**  **OR (95% CI)** |
| 9 months postpartum | | | | | |
| Vaginal birth | **1891** | **Ref** | **Ref** | **Ref** | **Ref** |
| Caesarean birth | **554** | **1.08 (1.00–1.20)** | **1.07 (0.93–1.20)** | **1.03 (0.92–1.16)** | **1.05 (0.93–1.17)** |
| 3 years postpartum | | | | | |
| Vaginal birth | **2650** | **Ref** | **Ref** | **Ref** | **Ref** |
| Caesarean birth | **750** | **1.03 (0.94–1.14)** | **1.05 (0.95–1.16)** | **1.00 (0.91–1.11)** | **1.03 (0.93–1.14)** |
| 5 years postpartum | | | | | |
| Vaginal birth | **3043** | **Ref** | **Ref** | **Ref** | **Ref** |
| Caesarean birth | **865** | **1.04 (0.95–1.15)** | **1.06 (0.96–1.17)** | **1.01 (0.92–1.12)** | **1.04 (0.94–1.15)** |
| 7 years postpartum | | | | | |
| Vaginal birth | **3335** | **Ref** | **Ref** | **Ref** | **Ref** |
| Caesarean birth | **928** | **1.01 (0.92–1.11)** | **1.03 (0.94–1.24)** | **0.97 (0.88–1.08)** | **1.03 (0.91–1.11)** |
| 11 years postpartum | | | | | |
| Vaginal birth | **3675** | **Ref** | **Ref** | **Ref** | **Ref** |
| Caesarean birth | **1023** | **1.01 (0.92–1.11)** | **1.04 (0.94–1.15)** | **0.98 (0.90–1.09)** | **1.01 (0.92–1.12)** |
| 14 years postpartum | | | | | |
| Vaginal birth | **3918** | **Ref** | **Ref** | **Ref** | **Ref** |
| Caesarean birth | **1104** | **1.04 (0.94–1.14)** | **1.07 (0.97–1.18)** | **1.00 (0.91–1.11)** | **1.05 (0.95–1.16)** |
| OR: Odd ratio, 95% CI: % Confidence interval, VB: Vaginal birth, CS: Caesarean section, BMI: Body mass index, HDP: Hypertensive disorders in pregnancy.  Model 1: Unadjusted  Model 2: Adjusted for maternal age, ethnicity, prepregnancy BMI.  Model 3: Adjusted for, Area deprivation level, maternal education, HDP, longstanding illness, parity.  Model 4: Fully adjusted.  *P-value <.05 | | | | | |
