# Supplementary material for: Mode of birth and maternal depression/severe anxiety: Findings from Millennium Cohort Study
Source: PLoS One. 2025 Jun 27;20(6):e0327129. doi: 10.1371/journal.pone.0327129 (PMC12204560; doi:10.1371/journal.pone.0327129)
Supplement: S3 Table — (DOCX) [file pone.0327129.s006.docx]

| S3 Table: Association between any induction and cumulative depression/severe anxiety at 9 months, 3,5,7,11, and 14 years postpartum among Millennium Cohort Study participants. | | | | | |
| --- | --- | --- | --- | --- | --- |
|  | **No of exposed cases** | **Model 1**  **OR (95% CI)** | **Model 2**  **OR (95% CI)** | **Model 3**  **OR (95%CI)** | **Model 4**  **OR (95% CI)** |
| At 9 months postpartum | | | | | |
| No induction | **1741** | **Ref** | **Ref** | **Ref** | **Ref** |
| Induced birth | **704** | **1.21 (1.10-1.34)*** | **1.17 (1.06-1.30)*** | **1.11 (1.00-1.23)*** | **1.09 (0.98-1.21)** |
| At 3 years postpartum | | | | | |
| No induction | **2443** | **Ref** | **Ref** | **Ref** | **Ref** |
| Induced birth | **957** | **1.19 (1.09-1.30)*** | **1.15 (1.04-1.26)*** | **1.09 (0.97-1.20)** | **1.07 (0.97-1.18)** |
| At 5 years postpartum | | | | | |
| No induction | **2813** | **Ref** | **Ref** | **Ref** | **Ref** |
| Induced birth | **1095** | **1.19 (1.09-1.31)*** | **1.15 (1.05-1.26)*** | **1.09 (0.99-1.20)** | **1.07 (0.97-1.18)** |
| At 7 years postpartum | | | | | |
| No induction | **3083** | **Ref** | **Ref** | **Ref** | **Ref** |
| Induced birth | **1180** | **1.17 (1.07-1.28)*** | **1.13 (1.03-1.23)*** | **1.07 (0.98-1.17)** | **1.05 (0.96-1.15)** |
| At 11 years postpartum | | | | | |
| No induction | **3399** | **Ref** | **Ref** | **Ref** | **Ref** |
| Induced birth | **1299** | **1.18 (1.08-1.29)*** | **1.14 (1.04-1.24)*** | **1.08 (0.99-1.19)** | **1.06 (0.97-1.16)** |
| At 14 years postpartum | | | | | |
| No induction | **3624** | **Ref** | **Ref** | **Ref** | **Ref** |
| Induced birth | **1398** | **1.21 (1.12-1.33)*** | **1.17 (1.07-1.28)** | **1.11 (1.02-1.22)*** | **1.09 (0.99-1.20)** |
| OR: Odd ratio, 95% CI: % Confidence interval, BMI: Body mass index, HDP: Hypertensive disorders in pregnancy.  Model 1: Unadjusted  Model 2: Adjusted for maternal age, ethnicity, prepregnancy BMI.  Model 3: Adjusted for, Area deprivation level, maternal education, HDP, longstanding illness, parity.  Model 4: Fully adjusted.  *P-value <.05 | | | | | |
