# Supplementary material for: Mode of birth and maternal depression/severe anxiety: Findings from Millennium Cohort Study
Source: PLoS One. 2025 Jun 27;20(6):e0327129. doi: 10.1371/journal.pone.0327129 (PMC12204560; doi:10.1371/journal.pone.0327129)
Supplement: S4 Table — (DOCX) [file pone.0327129.s007.docx]

| S4 Table: Association between mode of birth and cumulative depression/severe anxiety at 9 months, 3, 5,7, and 11 years postpartum among study participants with complete co-variates data (N=10456). | | | | | |
| --- | --- | --- | --- | --- | --- |
|  | No of exposed cases | Model 1  OR (95% CI) | Model 2  OR (95% CI) | Model 3  OR (95%CI) | Model 4  OR (95% CI) |
| Depression/severe anxiety diagnosis at 9 months postpartum | | | | | |
| Spontaneous VB | **1130** | **Ref** | **Ref** | **Ref** | **Ref** |
| Induced VB | **534** | **1.27 (1.13-1.43)*** | **1.21 (1.07-1.36)*** | **1.14 (1.01-1.29)*** | **1.10 (0.97-1.25)** |
| Assisted VB | **220** | **0.94 (0.80-1.11)** | **0.89 (0.76-1.05)** | **0.97 (0.72-1.15)** | **0.95 (0.80-1.04)** |
| Emergency CS | **169** | **1.03 (0.86-1.24)** | **1.02 (0.84-1.22)** | **0.99 (0.82-1.20)** | **1.01 (0.84-1.21)** |
| Planned CS | **214** | **1.21 (1.02-1.43)*** | **1.20 (1.01-1.43)*** | **1.12 (0.94-1.33)** | **1.10 (0.92-1.31)** |
| CS after Induction | **169** | **1.16 (0.97-1.40)** | **1.12 (0.92-1.35)** | **1.07 (0.88-1.30)** | **1.07 (0.88-1.31)** |
| Depression/severe anxiety diagnosis at 3 years postpartum | | | | | |
| Spontaneous VB | **1602** | **Ref** | **Ref** | **Ref** | **Ref** |
| Induced VB | **724** | **1.23 (1.10-1.37)*** | **1.17 (1.05-1.31)*** | **1.11 (1.00-1.25)** | **1.08 (0.96-1.21)** |
| Assisted VB | **314** | **0.94 (0.82-1.09)** | **0.90 (0.78-1.04)** | **0.99 (0.88-1.13)** | **0.95 (0.82-1.11)** |
| Emergency CS | **236** | **1.01 (0.85-1.19)** | **1.01 (0.85-1.19)** | **0.98 (0.83-1.16)** | **1.01 (0.85-1.20)** |
| Planned CS | **281** | **1.11 (0.95-1.29)** | **1.13 (0.96-1.32)** | **1.05 (0.89-1.23)** | **1.05 (0.89-1.23)** |
| CS after Induction | **231** | **1.12 (0.95-1.33)** | **1.09 (0.92-1.30)** | **1.04 (0.87-1.24)** | **1.06 (0.88-1.27)** |
| Depression/severe anxiety diagnosis at 5 years postpartum | | | | | |
| Spontaneous VB | **1823** | **Ref** | **Ref** | **Ref** | **Ref** |
| Induced VB | 834 | **1.27 (1.15-1.42)*** | **1.21 (1.09-1.35)*** | **1.15 (1.03-1.28)*** | **1.12 (1.00-1.25)*** |
| Assisted VB | 373 | **1.00 (0.87-1.15)** | **0.96 (0.84-1.11)** | **1.01 (0.87-1.17)** | **1.00 (0.87-1.17)** |
| Emergency CS | 279 | **1.07 (0.91-1.26)** | **1.07 (0.91-1.26)** | **1.03 (0.86-1.22)** | **1.06 (0.90-1.26)** |
| Planned CS | 326 | **1.15 (0.99-1.34)** | **1.18 (1.01-1.38)** | **1.10 (0.94-1.29)** | **1.10 (0.94-1.29)** |
| CS after Induction | 258 | **1.10 (0.93-1.30)** | **1.07 (0.91-1.27)** | **1.00 (0.84-1.19)** | **1.02 (0.85-1.22)** |
| Depression/severe anxiety diagnosis at 7 years postpartum | | | | | |
| Spontaneous VB | **2001** | **Ref** | **Ref** | **Ref** | **Ref** |
| Induced VB | 905 | **1.27 (1.14-1.41)*** | **1.21 (1.09-1.34)*** | **1.15 (1.03-1.28)*** | **1.10 (0.99-1.23)** |
| Assisted VB | 414 | **1.02 (0.89-1.17)** | **0.98 (0.85-1.13)** | **1.02 (0.88-1.18)** | **1.05 (0.90-1.21)** |
| Emergency CS | 298 | **1.03 (0.88-1.20)** | **1.03 (0.88-1.21)** | **0.98 (0.83-1.16)** | **1.04 (0.88-1.23)** |
| Planned CS | 356 | **1.15 (0.99-1.33)** | **1.19 (1.02-1.38)** | **1.11 (0.95-1.29)** | **1.13 (0.97-1.33)** |
| CS after Induction | 272 | **1.03 (0.88-1.21)** | **1.01 (0.86-1.20)** | **0.93 (0.78-1.10)** | **0.98 (0.83-1.18)** |
| Depression/severe anxiety diagnosis at 11 years postpartum | | | | | |
| Spontaneous VB | **2205** | **Ref** | **Ref** | **Ref** | **Ref** |
| Induced VB | 993 | **1.28 (1.16-1.42)*** | **1.22 (1.09-1.35)*** | **1.16 (1.04-1.29)*** | **1.12 (1.01-1.25)*** |
| Assisted VB | 457 | **1.03 (0.90-1.18)** | **0.98 (0.86-1.13)** | **1.02 (0.88-1.17)** | **1.01 (0.88-1.17)** |
| Emergency CS | 331 | **1.04 (0.89-1.22)** | **1.05 (0.90-1.23)** | **1.00 (0.85-1.17)** | **1.04 (0.88-1.22)** |
| Planned CS | 387 | **1.13 (0.98-1.31)** | **1.18 (1.01-1.37)*** | **1.10 (0.94-1.28)** | **1.11 (0.95-1.27)** |
| CS after Induction | 303 | **1.05 (0.90-1.24)** | **1.04 (0.88-1.22)** | **0.95 (0.80-1.12)** | **0.98 (0.82-1.16)** |
| Depression/severe anxiety diagnosis at 14 years postpartum | | | | | |
| Spontaneous VB | 2348 | **Ref** | **Ref** | **Ref** | **Ref** |
| Induced VB | 1058 | **1.30 (1.17-1.44)*** | **1.23 (1.11-1.37)*** | **1.18 (1.06-1.31)*** | **1.14 (1.02-1.27)*** |
| Assisted VB | 489 | **1.04 (0.91-1.19)** | **0.99 (0.87-1.14)** | **1.03 (0.90-1.19)** | **1.03 (0.89-1.19)** |
| Emergency CS | 357 | **1.07 (0.92-1.25)** | **1.08 (0.92-1.26)** | **1.03 (0.88-1.21)** | **1.08 (0.91-1.27)** |
| Planned CS | 407 | **1.11 (0.96-1.29)** | **1.17 (1.01-1.36)*** | **1.08 (0.93-1.26)** | **1.09 (0.94-1.28)** |
| CS after Induction | 337 | **1.15 (0.98-1.34)** | **1.14 (0.96-1.34)** | **1.04 (0.88-1.23)** | **1.08 (0.91-1.29)** |
| OR: Odd ratio, 95% CI: % Confidence interval, VB: Vaginal birth, CS: Caesarean section, BMI: Body mass index, HDP: Hypertensive disorders in pregnancy.  Model 1: Unadjusted  Model 2: Adjusted for maternal age, ethnicity, prepregnancy BMI.  Model 3: Adjusted for, Area deprivation level, maternal education, HDP, longstanding illness, parity.  Model 4: Fully adjusted.  *P-value <.05 | | | | | |
