# Supplementary material for: Mode of birth and maternal depression/severe anxiety: Findings from Millennium Cohort Study
Source: PLoS One. 2025 Jun 27;20(6):e0327129. doi: 10.1371/journal.pone.0327129 (PMC12204560; doi:10.1371/journal.pone.0327129)
Supplement: S5 Table — (DOCX) [file pone.0327129.s008.docx]

| S5 Table: Crude and adjusted association between mode of birth and cumulative depression/severe anxiety by 14 years postpartum-(only including participants at 14 years postpartum follow-up). | | | | | |
| --- | --- | --- | --- | --- | --- |
|  | No of exposed cases | Model 1  OR (95% CI) | Model 2  OR (95% CI) | Model 3  OR (95%CI) | Model 4  OR (95% CI) |
| Depression/severe anxiety diagnosis by 14 years postpartum | | | | | |
| Spontaneous VB | **1223** | **Ref** | **Ref** | **Ref** | **Ref** |
| Induced VB | **591** | **1.31 (1.16-1.47)*** | **1.23 (1.09-1.36)*** | **1.18 (1.04-1.33)*** | **1.13 (1.00-1.29)*** |
| Assisted VB | **280** | **1.09 (0.93-1.27)** | **1.04 (0.89-1.22)** | **1.07 (0.91-1.26)** | **1.07 (0.90-1.26)** |
| Emergency CS | **182** | **0.99 (0.83-1.19)** | **1.00 (0.83-1.21)** | **0.94 (0.78-1.15)** | **0.98 (0.81-1.19)** |
| Planned CS | **223** | **1.12 (0.95-1.33)** | **1.16 (0.97-1.38)** | **1.08 (0.91-1.29)** | **1.09 (0.91-1.30)** |
| CS after Induction | **199** | **1.34 (1.11-1.61)*** | **1.31 (1.09-1.58)*** | **1.24 (1.03-1.51)*** | **1.26 (1.03-1.54)*** |
| OR: Odd ratio, 95% CI: % Confidence interval, VB: Vaginal birth, CS: Caesarean section, BMI: Body mass index, HDP: Hypertensive disorders in pregnancy.  Model 1: Unadjusted  Model 2: Adjusted for maternal age, ethnicity, prepregnancy BMI.  Model 3: Adjusted for, Area deprivation level, maternal education, HDP, longstanding illness, parity.  Model 4: Fully adjusted.  *P-value <.05 | | | | | |
